# The nexus between improved water supply and water-borne diseases in urban areas in Africa: a scoping review protocol

**DOI:** 10.12688/aasopenres.13063.2

**Published:** 2020-12-08

**Authors:** Nyamai Mutono, James Wright, Henry Mutembei, Josphat Muema, Mair Thomas, Mumbua Mutunga, Samuel Mwangi Thumbi

**Affiliations:** 1 Wangari Maathai Institute for Peace and Environmental Studies, University of Nairobi, Nairobi, Kenya; 2 Washington State University Global Health – Kenya, Nairobi, Kenya; 3 Geography and Environmental Science, University of Southampton, Southampton, UK; 4 Department of Clinical Studies, Faculty of Veterinary Medicine, University of Nairobi, Nairobi, Kenya; 5 Institute of Tropical and Infectious Diseases, University of Nairobi, Nairobi, Kenya; 6 Paul G Allen School for Global Animal Health, Washington State University, Pullman, USA; 7 Institute of Immunology and Infection Research, University of Edinburgh, Edinburgh, UK; 8 NIHR Global Health Research Unit Tackling Infections to Benefit Africa (TIBA), University of Edinburgh, Edinburgh, UK

**Keywords:** Water-borne diseases, water insufficiency, scoping review, African cities, water supply

## Abstract

**Introduction**: Currently, an estimated two thirds of the world population is water insufficient. As of 2015, one out of every five people in developing countries do not have access to clean sufficient drinking water. In an attempt to share the limited resource, water has been distributed at irregular intervals in cities in developing countries. Residents in these cities seek alternative water sources to supplement the inadequate water supplied. Some of these alternative sources of water are unsafe for human consumption, leading to an increased risk in water-borne diseases. Africa contributes to 53% of the diarrheal cases reported globally, with contaminated drinking water being the main source of transmission. Water-borne diseases like diarrhea, cholera, typhoid, amoebiasis, dysentery, gastroenteritis, cryptosporidium, cyclosporiasis, giardiasis, guinea worm and rotavirus are a major public health concern. The main objective of this scoping review is to map the available evidence to understand the sources of water among residents in cities in Africa and the relationship between clean water sufficiency and water-borne diseases in urban Africa.

**Methods and analysis**: The search strategy will identify studies published in scientific journals and reports that are directly relevant to African cities that have a population of more than half a million residents as of 2014 AND studies on the ten emerging water-borne diseases, which are diarrhea, cholera, typhoid, amoebiasis, dysentery, gastroenteritis, cryptosporidium, cyclosporiasis, giardiasis, guinea worm and rotavirus.

**Ethics and dissemination: **This scoping review did not require any formal ethical approval. The findings will be published in a peer-reviewed journal.

## Introduction

Urbanization in sub-Saharan Africa (SSA) is growing at 4% annually, with the population living in urban areas projected to double by 2050 ^1^. This rapid urbanisation outpaces the development of infrastructure in these cities leading to inadequate access to basic amenities, including good housing, adequate social amenities, and continuous supply of safe drinking water to city residents ^2^. Poor access to clean water driven by rapid population growth, increased water demand, infrastructural constraints, and consumer response to cope with insufficient supply of safe water through use of alternative water sources is associated with ill-health ^3^. This rapid urbanisation is anticipated to accelerate demand for water ^2, 4^, which lies at the nexus of food security, poverty reduction, economic growth, energy production and human health ^5, 6^.

Currently, an estimated 40% of the global population is water insufficient ^7^. The World Health Organisation (WHO) international benchmark states a minimum water requirement per person per day of between 50 and 100 litres in order to meet basic domestic needs ^8^. However, by 2017, only half of the population residing in urban SSA had access to safely managed drinking water that was free from contamination. Goal 6 of the Sustainable Development Goals (SDGs) aims to attain sustainable management and availability of sufficient clean water and sanitation for all ^7^. This aligns with Aspiration 1 of the African Union Agenda 2063 objectives, on sustainable development in Africa ^9^.

Residents of urban areas in Africa depend on both improved and unimproved water sources including piped water, boreholes, wells, vendors and surface water ^8^. However, the Africa Infrastructure Country Diagnostic report by the World Bank categorised piped water as the only major source of improved water in SSA ^10^. In 2017, piped water was only accessible to 230 million people (61%) in urban areas in this region ^11^. In an attempt to share and ration limited resources, piped water has been distributed in cities in developing countries at irregular intervals, known as intermittent water supplies (IWS) ^12^. Residents have responded to these challenges by seeking alternative sources of water, some of which are unsafe for human consumption ^13, 14^. Half (53%) of the water sources in Africa are faecally contaminated, predisposing the people to the risk of diarrheal diseases ^15^.

In 2016, more than half a billion deaths in SSA were attributed to diarrheal diseases with contamination of drinking water identified as one of the leading risk factors ^16^. Major pathogens, such as *Escherichia coli*, cryptosporidium, *aeromonas* spp, shigella and entamoeba, often found in unsafe water, are associated with moderate-to-severe diarrhea which is especially life threatening to infants ^17^. Disease surveillance and response in SSA lists cholera, diarrheal diseases and typhoid fever as some of the priority diseases associated with poor quality water, that should be regularly monitored and reported ^18^.

Previous reviews have explored the prevalence of intermittent water supplies in low income settings ^19^, household water availability across Africa ^20^, the global burden of diarrheal diseases ^16, 21^ and implications of intermittent water supply on gastrointestinal illness ^22^. We have found no review focused on urban areas in Africa and the implications of water-borne diseases as a result of intermittent piped water in this region.

This manuscript details the protocol to a review of the link between insufficient piped water supply and waterborne diseases and syndromes in urban Africa. In doing so, it seeks to address the following research question: “what is the proportion of residents with safely managed water in cities in Africa and what is the correlation with water-borne diseases and the symptoms?” This will be achieved by synthesising findings of studies on: a) water sufficiency in cities within Africa; b) consequences of rapid urbanisation on water sufficiency in African cities; and c) the linkages between water sufficiency and water-borne diseases and their symptoms in Africa.

This work should provide information to guide policies that aim to help Africa achieve one of its Agenda 2063 aspirations on provision of adequate basic necessities for urban populations in the region ^9^.

### Definitions

For the purpose of this protocol and the planned review, key terms are defined and classified as follows:

• **Waterborne disease:** includes cholera, typhoid, amoebiasis, cyclosporiasis and giardiasis diseases ^16– 18^

• **Symptoms of waterborne diseases:** focus on diarrhea, dysentery and gastroenteritis ^16, 17^

• **Etiological agents of diarrheal diseases:** include cryptosporidium and rotavirus ^16, 17^

• **Water insufficiency:** is classified as having less than 50 litres per person per day ^8^

• **City:** an urban area with a population of more than half a million residents ^23^

## Methods

The scoping review will use the Joanna Briggs Institute methodology guidance ^24^ and the Preferred Reporting Items for Systematic Reviews and Meta-Analyses (PRISMA) guidelines for conducting systematic reviews and meta-analysis ( http://www.prisma-statement.org). These methodologies have been used for published scoping reviews ^25, 26^.

### Inclusion criteria

**Figure 1.**
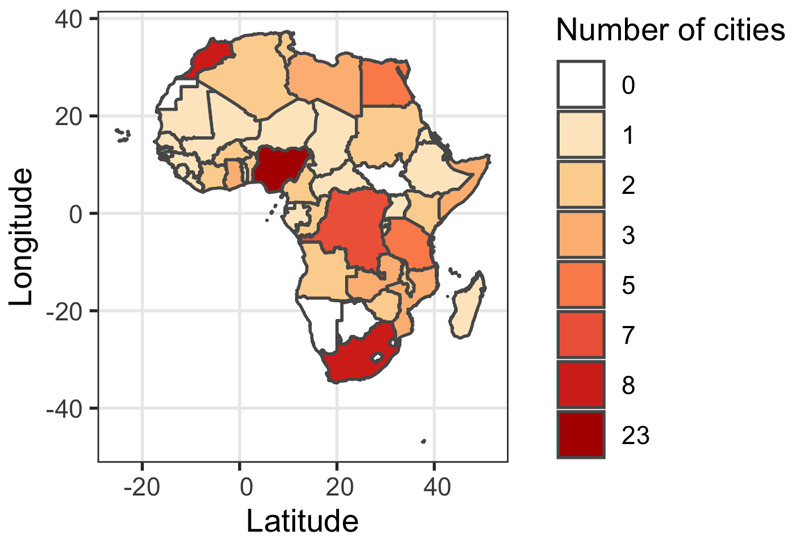
Member countries in the African Union and the number of cities with a population greater than half a million residents. Source of data: United Nations World Urbanisation Prospects, 2014 ^27^.

The review will include the following criteria:

1)    Studies undertaken in African Union member states.

2)    Studies describing the water situation in cities, classified as urban areas with greater than half a million residents. Since the classification of a city and urban environments is not standardised ^2^, we use areas with a population greater than 0.5 million people to be consistent with the UN report that estimates one in every three people will reside in cities with at least half a million inhabitants by 2030 ^23^ ( Figure 1). The list of the cities that meet this criterion have been selected from the United Nations World Urbanisation Prospects of 2014 ^27^.

3)    Studies focusing on water-borne diseases, symptoms and etiological agents:

a)    Diseases include cholera, typhoid, amoebiasis, cyclosporiasis and giardiasis diseases

b)    Symptoms include diarrhea, dysentery and gastroenteritis

c)    Etiological agents include cryptosporidium and rotavirus

4)    Studies published in scientific journals or grey literature from government or non-governmental organisations

### Exclusion criteria

1)    Studies not written in the English or French language

2)    Systematic reviews

3)    Studies conducted in non-member states of the African Union

### Search strategy

Comprehensive literature searches will be undertaken in Embase, MEDLINE, Web of Science and Google Scholar databases. These four databases have been identified as the optimal combination of databases that will guarantee adequate coverage of studies for this scoping review ^28^.

The search strategy will take a three-step process. The first step will involve carrying out a limited search in MEDLINE, Google Scholar (first 500 results), Embase and Web of Science databases. The text and index terms that are used to describe the articles will be assessed. The second step will include searches using the keywords and index terms. In the final step, we will go through the references to identify key articles that might have been missed in the first two steps. The search terms used in the study are seen in Table 1.

**Table 1.** Search terms that will be used to identify studies.

Parameter	Search terms
Population	Huambo OR Luanda OR Cotonou OR “Abomey-Calavi” OR “Abomey Calavi” OR Ouagadougou OR Bobo-Dioulasso OR “Bobo Dioulasso” OR Bunjumbura OR Younde OR Yaounde OR Douala OR Bangui OR Ndjamena OR Brazaville OR Pointe-Noire OR “PointeNoire” OR Abidjan OR Bouake OR Kinsasha OR Cairo OR “Al Qahirah” OR Al-Qahirah OR Alexandria OR “Al-Iskandariyah” OR “Al Iskandariyah” OR “Port Said” OR “Bur Said” OR “Addis Ababa” OR Libreville OR Banjul OR Accra OR Kumasi OR Conakry OR Nairobi OR Mombasa OR Monrovia OR Antananarivo OR Lilongwe OR “Blantyre-Limbe” OR “Blantyre Limbe” OR Bamako OR Nouakchott OR Casablanca OR “Dar-el-Beida” OR “Dar el Beida” OR Rabat OR Nampula OR Tetouan OR Fes OR Marrakech OR Tangier OR Tanger OR Maknes OR Meknes OR Agadir OR Maputo OR Matola OR Niamey OR Lagos OR Kaduna OR Akure OR Kano OR Abuja OR Aba OR Kigali OR Dakar OR Freetown OR CapeTown OR Durban OR Pretoria OR “Port Elizabeth” OR Bloemfontein OR “Dar es Salaam” OR Arusha OR Mbeya OR Lome OR Kampala OR Kitwe OR Lusaka OR Harare OR Bulawayo OR “Benin City” OR Enugu OR Ibadan OR Ikorodu OR Ilorin OR Jos OR Maiduguri OR Nnewi OR Onitsha OR Oshogbo OR Owerri OR “Port Harcourt” OR Sokoto OR Umuahia OR Oyo OR Warri OR Zaria OR Hargeysa OR Merca OR Mogadishu OR Muqdisho OR Johannesburg OR Soshanguve OR Vereeniging OR Khartoum OR “Al-Khartum” OR “Al Khartum” OR Nyala OR Safaqis OR Tunis OR Mwanza OR Zanzibar OR Ndola OR Algiers OR “El Djazair” OR Wahran OR Oran OR Bukavu OR Kananga OR Kisangani OR Lubumbashi OR “Mbuji-Mayi” OR “Mbuji Mayi” OR Tshikapa OR Djibouti OR “Al-Mansurah” OR “Al Mansurah” OR “As-Suways” OR “As Suways” OR Asmara OR “Sekondi Takoradi” OR Banghazi OR Misratah OR Tarabulus OR Tripoli
	*AND*
Exposure	water AND (scarc* OR intermittent OR break* OR ratio* OR deficit OR deficien* OR unavailab* OR continu* OR interrupt* OR stress OR supply OR sufficien* OR insufficien*)
	*AND*
Outcome	“water borne” OR “water-borne” OR cholera OR typhoid OR diarrhea* OR diarrhoea OR amoebiasis OR dysentery OR gastroenteritis OR cryptosporidi* OR cyclosporiasis OR giardiasis OR rotavirus

### Study selection

Once the searches have been undertaken in the databases, the title and abstracts will be extracted from the articles. Duplicates will be removed, and the review team will screen the studies using two levels: initial screening and full-text screening. During the initial screening process, three reviewers will read the abstracts of the studies captured by the search terms and assess their relevance in light of the inclusion criteria. To ensure consistency, 10% of all the studies will be randomly selected and independently reviewed by one other reviewer. Any inconsistencies between the primary and secondary reviewers will be discussed and a consensus reached.

Full text articles will be obtained for the studies that pass the initial screening stage. Microsoft Excel (version 16.36) will be used to store the extracted data. Table 2 shows the characteristics that will be extracted from each study. Of the data extracted, 10% will be randomly selected and independently reviewed by one other reviewer. Any inconsistencies amongst the reviewers will be discussed and an agreement will be reached.

**Table 2.** Variables to be extracted from the articles for full-text screening.

	Variable	Details
1	Authors	Authors of the article
2	Publication type	Thesis, article
3	Title of the article	Full title
4	Year of publication	Year the article was published or written
5	Geographical scope of the study	City/cities the study was conducted
6	Study type	
7	Duration of the study (if applicable)	
8	Rate of urbanisation	Metric, population of the city
9	Water demand/supply	Main water source, main water distributor, water demand
10	Indicators of water supply	Frequency of water supply, water rationing, cost, coverage, quality
11	Water-borne diseases/symptoms/etiological agents	Diarrhoea, cholera, typhoid, amoebiasis, dysentery, gastroenteritis, cryptosporidium, cyclosporiasis, giardiasis, rotavirus
12	Cases of water-borne disease/symptoms/etiological agents	Lab-confirmed/self-reported/clinically diagnosed
13	Water insufficiency	Metric, proportion of urban population with sufficient water supply, proportion of urban population with insufficient water supply
14	Use of the WHO water insufficiency classification of less that 50 litres per person per day	Yes/No
15	Proportion of population with water borne diseases	Metric
16	Area proposed for future research	

All irrelevant studies will be removed and the reason for their exclusion will be recorded. In this stage, another 10% of the studies will be sampled and shared with the secondary reviewer who will exclude or include the studies based on their relevance to the study objective. Consensus will be reached for any discrepancies in the studies among the reviewers.

## Presentation of results

If a sufficient number of studies report on the effect of water insufficiency on health outcomes, we will calculate heterogeneity (I ^2^) for this subset. The index of heterogeneity (I ^2^ statistic) will be calculated from the sum of the squared deviations of the estimate of each study, from the overall estimate, and weighted by the influence of the study on the calculation of the overall estimate. We will examine the risk bias in the study level and characterize whether the metrics of water insufficiency and health are representative of the whole urban population or only a sub-group. We will use the R statistical software (version 3.6.1) to conduct this analysis ^29^.

Cluster analysis will be performed to collate similar studies using Ward’s agglomerative hierarchical clustering method, which is used in other scoping reviews ^30^. The optimal number of clusters will be chosen to ensure the inner homogeneousness and external heterogeneousness of a cluster is balanced. For studies that focus on diarrheal disease, we will differentiate the self-reported studies from those with etiological characterisation of pathogens and input these studies into the planned cluster analysis.

The study locations will be geo-coded and the data will be presented using digital maps that will depict the water sufficiency in these different cities. The results will be linked to the coordinates from the peer reviewed and publicly available water scarcity map layer from the Water Footprint Network ^31^ which has been used in previous systematic reviews ^32^. This will allow us to make observations on the trend between cities with prevalence of waterborne diseases and water scarcity. The resultant maps will enable researchers to identify areas that have gaps in knowledge and research needs.

### Ethics and dissemination

The study does not involve any interviews or interactions with humans or animals and does not require ethical approval. The findings will be published in a scientific peer-reviewed journal.

### Study status

Currently, we are undertaking the literature searches in the MEDLINE, Embase, Web of Science and Google Scholar databases and extracting the titles and abstracts from the articles which will be used in the initial screening process.

## Data availability

No data are associated with this article.
