# Supplementary material for: The nexus between improved water supply and water-borne diseases in urban areas in Africa: a scoping review protocol
Source: AAS Open Res. 2020 Dec 8;3:12. Originally published 2020 May 5. [Version 2] doi: 10.12688/aasopenres.13063.2 (PMC7883317; doi:10.12688/aasopenres.13063.2)
Supplement: Peer review history

# Peer review: Reviewer response for version 2

**DOI:** 10.21956/aasopenres.14295.r28241

**Published:** 2021-02-12

**Authors:** Batsirai Majuru

**Affiliations:** 1 World Health Organization, Geneva, Switzerland

This is a much improved protocol.

- On the definition of water insufficiency, the authors may wish to note that the WHO document cited by Gleick (reference #8 in the manuscript) has recently been updated, and the main recommendations are 5.3 L/person/day for drinking, 20 L/person/day for basic access and a national policy for intermediate access at least 50 L/person/day. 
- The drinking-water target ensures hydration for lactating women engaged in moderate activity at moderately high temperatures – the population group with the highest physiological needs.
- Basic access is needed to ensure sufficient qualities of water are available for drinking, cooking, food hygiene and handwashing and face washing under most circumstances. 
- A national policy objective of at least intermediate access of 50 L/person/day is recommended to ensure adequate quantities of water are available for drinking, cooking, food hygiene and all personal hygiene (handwashing, face washing, bathing and laundry), under most circumstances

Is the study design appropriate for the research question?

Partly

Is the rationale for, and objectives of, the study clearly described?

Partly

Are sufficient details of the methods provided to allow replication by others?

Partly

Are the datasets clearly presented in a useable and accessible format?

Not applicable

Reviewer Expertise:

Public health, water quality, water policy and regulation

I confirm that I have read this submission and believe that I have an appropriate level of expertise to confirm that it is of an acceptable scientific standard.

## References

[1] : Domestic water quantity, service level and health. *World Health Organization*.2020; Reference source

# Peer review: Reviewer response for version 2

**DOI:** 10.21956/aasopenres.14295.r28242

**Published:** 2021-01-08

**Authors:** David Musoke

**Affiliations:** 1 Department of Disease Control and Environmental Health, School of Public Health, Makerere University College of Health Sciences, Kampala, Uganda

The authors have addressed most of the comments raised earlier. However, the introduction of the abstract can further be made more succinct, and statistics omitted as suggested earlier. In addition, the earlier concern regarding the exclusion criteria has not been satisfactorily addressed.

Is the study design appropriate for the research question?

Yes

Is the rationale for, and objectives of, the study clearly described?

Yes

Are sufficient details of the methods provided to allow replication by others?

Partly

Are the datasets clearly presented in a useable and accessible format?

Not applicable

Reviewer Expertise:

NA

I confirm that I have read this submission and believe that I have an appropriate level of expertise to confirm that it is of an acceptable scientific standard.

# Peer review: Reviewer response for version 1

**DOI:** 10.21956/aasopenres.14158.r27733

**Published:** 2020-08-24

**Authors:** David Musoke

**Affiliations:** 1 Department of Disease Control and Environmental Health, School of Public Health, Makerere University College of Health Sciences, Kampala, Uganda

Generally, this is a well written protocol for the scoping review which is likely to generate key information concerning water and related diseases in cities in Africa. However, my main concern as detailed below is the need to explore other water indicators beyond sufficiency as they also contribute to water borne diseases.

**Abstract:**

- The background in the abstract is very wordy and therefore can be made more succinct.
- Use of statistics in the background of an abstract is discouraged due to the inability to cite accordingly.

 

**Background:**

- Whereas the background has focused on water sufficiency (quantity), it is important for the authors to consider describing other key water indicators such as coverage, quality, cost and continuity that also contribute to the occurrence of water borne diseases.
- The background may be strengthened by providing information on the various sources of water used in urban settings in Africa, both improved and unimproved.
- Water statistics for 2015 are used yet more recent literature is available.
- Whereas the study aim is on water sufficiency, other parameters as noted above have a direct contribution to water borne diseases. It is therefore not clear how these parameters are to be considered in the scoping review.

 

**Protocol:**

- In the inclusion criteria, other water indicators noted above should be considered.
- The exclusion criteria in principle should not be the opposite of the inclusion criteria but rather any predefined conditions that will be used omit any studies that would have met the inclusion criteria.
- The exposure search terms as well as variables (Table 2) may also include the various water indicators beyond quantity.
- The use of the term ‘emerging diseases’ may need to be justified.
- The choice of selection of the 10 ‘diseases’ also needs to be justified.
- The outcome and independent variables to be considered in the review need to be described explicitly in the protocol.

Is the study design appropriate for the research question?

Yes

Is the rationale for, and objectives of, the study clearly described?

Yes

Are sufficient details of the methods provided to allow replication by others?

Partly

Are the datasets clearly presented in a useable and accessible format?

Not applicable

Reviewer Expertise:

Environmental and Public Health including water, sanitation and hygiene.

I confirm that I have read this submission and believe that I have an appropriate level of expertise to confirm that it is of an acceptable scientific standard, however I have significant reservations, as outlined above.

# Response

**Published:** 2020-12-03

**Authors:** Mutono Nyamai

**Affiliations:** University of Nairobi, Kenya

*The background in the abstract is very wordy and needs to be more succint*

- We have substantially shortened the ‘background’ section of the abstract in order to make it more succinct.

*Use statistics in the background of an abstract is discouraged*

- We have removed statistics from the background section of the abstract.

*It is important for the authors to consider describing other key water indicators such as coverage, quality, cost and continuity that also contribute to the occurrence of water borne diseases.*

- In order to limit the scope of this review we have specifically focused on insufficiency, as well as large African cities with population of more than 0.5 million AND selected waterborne diseases/symptoms/etiological agents AND water situation. In order to address other water indicators like cost, coverage, etc.  and how they may be possible confounders, we will extract this information from the articles during the full text screening process as highlighted in Table 2, point 10. 

*The background may be strengthened by providing information on the various sources of water used in urban settings in Africa, both improved and unimproved.*

- We agree with the reviewer and have therefore edited line 60 so that it now includes the main sources of water used in urban areas in the African content.

*Water statistics for 2015 are used yet more recent literature is available.*

- Paragraph 2 and 3 have been updated to include the recent statistics from the UNICEF/WHO Joint Monitoring Program report. 

*Whereas the study aim is on water sufficiency, other parameters as noted above have a direct contribution to water borne diseases. It is therefore not clear how these parameters are to be considered in the scoping review.*

- These parameters have been included in Table 2 which highlights the variables that will be extracted from the articles during the full-text characterisation process. We will report how they contribute to waterborne diseases.

*In the inclusion criteria, other water indicators noted above should be considered.*

- Point 10, Table 2 lists the other indicators which will be extracted from the articles during the full-text screening process. 

*The exclusion criteria in principle should not be the opposite of the inclusion criteria but rather any predefined conditions that will be used omit any studies that would have met the inclusion criteria.*

- We agree with the reviewer and have therefore modified the exclusion criteria and only listed conditions that will omit studies that meet the inclusion criteria.

*The exposure search terms as well as variables (Table 2) may also include the various water indicators beyond quantity.*

- Point 10 of Table 2 has been updated to include other indicators of water supply (frequency of water supply, water rationing, cost, coverage, quality).

*The use of the term ‘emerging diseases’ may need to be justified.*

- Based on the advice from the first reviewer, this term has been omitted from the protocol. 

*The choice of selection of the 10 ‘diseases’ also needs to be justified*

- We have modified this to exclude ‘10 diseases’. The ‘Definitions’ section now classifies waterborne diseases, symptoms and etiological agents of diarrheal diseases, and includes justifications where appropriate.

*The outcome and independent variables to be considered in the review need to be described explicitly in the protocol.*

- As this is a scoping review rather than a systematic review, we will focus on the methods of the included studies, as opposed to their findings or examination of effect sizes derived for outcomes variables. This will form the inputs to the cluster analysis, so we do not have an outcome and an independent variable given the nature of the type of review we will be conducting.

# Peer review: Reviewer response for version 1

**DOI:** 10.21956/aasopenres.14158.r27452

**Published:** 2020-05-29

**Authors:** Batsirai Majuru

**Affiliations:** 1 World Health Organization, Geneva, Switzerland

The paper outlines a protocol for a scoping review of the links between sufficiency of water supply and water-borne diseases in cities in the African region. In the protocol, the authors propose to conduct a search of both peer reviewed and grey literature on water sufficiency in African cities of >500,000 residents and 'ten emerging water-borne diseases'.

 The authors attempt to address pertinent questions regarding water supply in the African region. However, the protocol in its current state reads like a decent initial draft, that now requires refinement and sharpening. There are several areas that are unclear. I have tried to offer what I hope is useful criticism.

**The rationale for, and objectives of the study are somewhat unclear.**

 Per the protocol, the question to be answered is: 'what is the water sufficiency in cities in Africa and what is the correlation with water-borne diseases?' There are several concerns with the scope, definition of terms, and assessment criteria that make the rationale and objectives of the review somewhat unclear. These are outlined below.

**The study design is not entirely appropriate for the research question.**

 The protocol requires further development and elaboration, primarily in the outcome variables of interest i.e. the waterborne diseases. The central idea in the protocol seems to be linking water insufficiency to the 'ten emerging waterborne diseases'. There are several concerns here. 

1. The terminology needs to be clarified and standardized: 
  -Diarrhoea is a symptom of several diarrhoeal diseases, including cholera and typhoid. Classifying it as a disease in itself is inaccurate.
  -Gastroenteritis is a set of symptoms (including diarrhoea, vomiting, nausea) arising from intestinal infection, so again, classifying it as a disease in itself is inaccurate.
  - *Cryptosporidium *and rotavirus are microorganisms / aetiological agents that cause diarrhoeal diseases, but are not diseases in themselves.
  -Guinea worm is a parasite, not a disease in itself. Diarrhoea is not a typical symptom of dracunculiasis (guinea worm disease).
2. What criteria were used to classify the 'emerging' diseases? This should be clearly described.
  - The question whether the diseases / symptoms / infectious agents listed in the protocol are 'emerging' is highly debatable. The paper cited on some of the said emerging diseases is from 16 years ago - it is fair to say that the landscape has changed a lot since then. 
  - The GEMS 2013 study on aetiology of moderate-severe diarrhoea in low-income countries highlighted rotavirus, ETEC, *Cryptosporidium* and *Shigella* as the main pathogens of concern. There are likely more recent studies on this, which the authors are advised to look up.
  - Guinea worm disease / dracunculiasis has been eradicated in most countries, with about 30 cases per year (sometimes less) now reported from 3 or 4 countries in Africa, so it is unclear how it could be termed an emerging disease.
3. If the hypothesis is that water insufficiency leads to waterborne disease, how is the insufficiency determined? E.g. if City X has a published water rationing schedule in which water is supplied 12 hours per day for 7 days a week, vs City Y that intermittently supplies water 2 days a week - are the households in both cities water insufficient? I have no bright ideas on this, but it may be food for thought.
4. Most diarrhoeal cases in literature are self-reported, thus aetiological info will likely be limited.
  - Is there some weighting that would be assigned to studies based on the depth of information on the waterborne disease(s) provided? Studies reporting on the aetiology of the diarrhoeal disease may arguably carry more weight than those only reporting diarrhoea / gastroenteritis, which may not necessarily be waterborne.

**Some definition of terms and scope is required for the review to be replicated by others.**

 It is unclear how 'water sufficiency' is defined within the scope of the review. E.g. for households living in slums, water insufficiency may arise from the slum not being connected to the network. Are they within the scope of the review?

 Water deficiency, water scarcity and water insufficiency are used throughout the protocol. Do these terms all have the same meaning within the context of the review?

 Other comments:

1. What is the reference for the 53% of diarrhoeal cases being from Africa? The paper by Bain *et al.* cited as ref #13 actually reports 53% of water sources in Africa being faecally contaminated, but does not refer to this 53% as the diarrhoeal disease burden from the African region.
2. Table 2, variable 14 lists 'consumer' under 'Main source of water scarcity metric'. What does this mean? How are consumers sources of water scarcity?
3. Water scarcity is not the only reason for intermittent supply. See for example review by Galaitsi *et al.* (2016 ^1^).
4. In the abstract, sentence 2: The 2015 statistic is not static, so somewhat inaccurate to quote as: 'As of 2015, xx people do not have access to safe drinking water'. Actually, the most recent estimate is that 1 in 3 do not have access to safe drinking-water. 

Is the study design appropriate for the research question?

Partly

Is the rationale for, and objectives of, the study clearly described?

Partly

Are sufficient details of the methods provided to allow replication by others?

Partly

Are the datasets clearly presented in a useable and accessible format?

Not applicable

Reviewer Expertise:

Public health, water quality, water policy and regulation

I confirm that I have read this submission and believe that I have an appropriate level of expertise to confirm that it is of an acceptable scientific standard, however I have significant reservations, as outlined above.

## References

[1] : Intermittent Domestic Water Supply: A Critical Review and Analysis of Causal-Consequential Pathways. *Water*.2016;8(7) : 10.3390/w8070274 10.3390/w8070274

# Response

**Published:** 2020-12-03

**Authors:** Mutono Nyamai

**Affiliations:** University of Nairobi, Kenya

*The terminology needs to be clarified and standardised:*

*-Diarrhoea is a symptom of several diarrhoeal diseases, including cholera and typhoid. Classifying it as a disease in itself is inaccurate.*

*-Gastroenteritis is a set of symptoms (including diarrhoea, vomiting, nausea) arising from intestinal infection, so again, classifying it as a disease in itself is inaccurate.*

*-Cryptosporidium and rotavirus are microorganisms / etiological agents that cause diarrhoeal diseases but are not diseases in themselves.*

**

*-Guinea worm is a parasite, not a disease in itself. Diarrhoea is not a typical symptom of dracunculiasis (guinea worm disease).*

- In lines 94 – 103, we have clarified the terminology and improved the classification of waterborne diseases, symptoms and etiological agents as follows:
  -  
**Waterborne disease: **includes cholera, typhoid, amoebiasis, cyclosporiasis and giardiasis diseases.
  - **Symptoms of waterborne diseases:** focus on diarrhea, dysentery and gastroenteritis.
  - **Etiological agents of diarrheal diseases:** include cryptosporidium and rotavirus.
- We added a key definitions section after the introduction which aims to clarify any ambiguity and standardise the terminology.
- We also removed guinea worm from our search strategy in Table 1 (line 140).

*What criteria were used to classify the 'emerging' diseases? This should be clearly described.*

*- The question whether the diseases / symptoms / infectious agents listed in the protocol are 'emerging' is highly debatable. The paper cited on some of the said emerging diseases is from 16 years ago - it is fair to say that the landscape has changed a lot since then.*

*- The GEMS 2013 study on etiology of moderate-severe diarrhoea in low-income countries highlighted rotavirus, ETEC, Cryptosporidium and Shigella as the main pathogens of concern. There are likely more recent studies on this, which the authors are advised to look up.*

**

*- Guinea worm disease / dracunculiasis has been eradicated in most countries, with about 30 cases per year (sometimes less) now reported from 3 or 4 countries in Africa, so it is unclear how it could be termed an emerging disease.*

- We have removed the terminology “emerging diseases” as the reference was outdated and its use as a term was ambiguous, as highlighted by the reviewer. In turn, we have changed the emphasis of the paper to mainly focus on the diseases/symptoms/ etiological agents. As mentioned, we specifically define these in the ‘Definitions’ section in lines 89-99.
- We looked at the GEMS 2013 and GEMS 2019 study which highlighted cryptosporidium, enteropathogenic *Escherichia, *shigella and rotavirus as the main pathogens of concern. We decided to focus on rotavirus and cryptosporidium in our scoping review. Globally, rotavirus was the leading etiology for diarrhea mortality among all ages[1] while cryptosporidium had the highest number of deaths with the pathogen present in children between 12-23 months[2].
- We agree with the reviewer that dracunculiasis is not an emerging disease as it has been eradicated in most countries hence it has been removed from our inclusion criteria.

*If the hypothesis is that water insufficiency leads to waterborne disease, how is the insufficiency determined? E.g. if City X has a published water rationing schedule in which water is supplied 12 hours per day for 7 days a week, vs City Y that intermittently supplies water 2 days a week - are the households in both cities water insufficient? I have no bright ideas on this, but it may be food for thought.*

- In this scoping review, the term ‘water insufficiency’ relates to water quantity, where we used the World Health Organisation’s (WHO) international benchmark of less than 50 litres of piped water per person per day[3], as highlighted in lines 49-51. We also included the definition of water insufficiency as used in this paper on line 102.
- In order to address instances such as that which the reviewer gives as an example, we will record how water insufficiency was assessed in the papers and if there are enough articles that use methods for characterising water insufficiency, a categorical variable depicting the methods used will form an input within the cluster analysis . We highlight this in point 13 of Table 2.

*Most diarrhoeal cases in literature are self-reported, thus etiological info will likely be limited.*

*- Is there some weighting that would be assigned to studies based on the depth of information on the waterborne disease(s) provided? Studies reporting on the etiology of the diarrhoeal disease may arguably carry more weight than those only reporting diarrhoea / gastroenteritis, which may not necessarily be waterborne.*

- In order to address the concern raised by the reviewer on majority of the studies on diarrheal cases being self reported, we will differentiate studies with self-reports from those with etiological characterisation of pathogens. The differences will then be inputted into the planned cluster analysis of study methods (see lines 173-178).  

*It is unclear how 'water sufficiency' is defined within the scope of the review. E.g. for households living in slums, water insufficiency may arise from the slum not being connected to the network. Are they within the scope of the review?*

*Water deficiency, water scarcity and water insufficiency are used throughout the protocol. Do these terms all have the same meaning within the context of the review?*

- We have now specifically outlined how we define water insufficiency in the new ‘Definitions’ section (line 93). We will use the WHO definition of less than 50 litres per person per day as water insufficiency (line 102). One of the variables extracted from the included studies is the use of the WHO water insufficiency definition, as highlighted in Table 2 point 14.
- Informal settlements are within the scope of this review. Water is often distributed in informal settlements through water kiosks or public taps[4]. Study setting (informal versus formal) would be included as a study characteristic within cluster analysis, to help examine if different methods and study designs are adopted in such settings.  
- We agree with the reviewer that water scarcity, water deficiency and water insufficiency were used throughout the protocol and to remove any ambiguity, we will focus on water insufficiency but include water scarcity and water deficiency in our search terms to ensure we do not miss out any articles on water insufficiency.

*What is the reference for the 53% of diarrhoeal cases being from Africa? The paper by Bain et al. cited as ref #13 actually reports 53% of water sources in Africa being faecally contaminated, but does not refer to this 53% as the diarrhoeal disease burden from the African region.*

- We have removed this reference and restructured the sentence (see lines 68-69).

*Table 2, variable 14 lists 'consumer' under 'Main source of water scarcity metric'. What does this mean? How are consumers sources of water scarcity?*

- The term “consumer” was removed from point 14 in Table 2. 

*Water scarcity is not the only reason for intermittent supply. See for example review by Galaitsi et al. (2016).*

- We agree with the reviewer that water scarcity is not the only reason for intermittent supply. We have addressed this issue in lines 46-49, which captures the main drivers of insufficient water supply as reported by Galaitsi et al[5].

*In the abstract, sentence 2: The 2015 statistic is not static, so somewhat inaccurate to quote as: 'As of 2015, xx people do not have access to safe drinking water'. Actually, the most recent estimate is that 1 in 3 do not have access to safe drinking-water.*

- Based on the second reviewer’s comment about removing statistics from the abstract, this statement has been removed. 

[1] Estimates of the global, regional and national morbidity, mortality and aetiologies of diarrhoea in 195 countries: a systematic analysis for the Global Burden of Disease Study 2016 https://www.thelancet.com/journals/laninf/article/PIIS1473-3099(18)30362-1/fulltext

 [2] Diarrhoeal disease and subsequent risk of death in infants and children residing in low-income and middle-income countries: analysis of the GEMS case-control study and 12-month GEMS-1A follow-on study https://www.thelancet.com/journals/langlo/article/PIIS2214-109X(19)30541-8/fulltext

 [3] Basic Water Requirements for Human Activities: Meeting Basic Needs https://www.tandfonline.com/doi/abs/10.1080/02508069608686494?journalCode=rwin20

 [4] Informal water vendors and the urban poor https://pubs.iied.org/pdfs/10529IIED.pdf

 [5] Intermittent domestic water supply: A critical review and analysis of causal-consequential pathways https://www.mdpi.com/2073-4441/8/7/274
